# A novel *PTEN* variant causing hemimegalencephaly and focal nodular heterotopias in the developing human brain

**DOI:** 10.1002/epi.70088

**Published:** 2026-01-05

**Authors:** Franziska Fazekas, Amit Haboosheh, Bernhard Hennebichler, Thomas Roetzer‐Pejrimovsky, Julia Binder, Theresa Reischer, Mateja Pfeifer, Anke Scharrer, Christof Worda, Tina Linder, Alex Farr, Romana Höftberger, Ellen Gelpi, Christian Mitter, Gregor Kasprian, Christine Haberler, Nicole Amberg

**Affiliations:** ^1^ Department of Neurology, Division of Neuropathology and Neurochemistry Medical University of Vienna Vienna Austria; ^2^ Comprehensive Center for Clinical Neurosciences and Mental Health Medical University of Vienna Vienna Austria; ^3^ Department of Biomedical Imaging and Image‐Guided Therapy, Division of Neuroradiology and Musculoskeletal Radiology Medical University of Vienna Vienna Austria; ^4^ Department of Radiology Hadassah Medical Center Jerusalem Israel; ^5^ Department of Obstetrics and Gynecology, Division of Obstetrics and Feto‐Maternal Medicine, and Comprehensive Center for Pediatrics Medical University of Vienna Vienna Austria; ^6^ Medical University of Vienna Center for Pathobiochemistry and Genetics, Institute of Medical Genetics Vienna Austria; ^7^ Department of Pathology Medical University of Vienna Vienna Austria

**Keywords:** brain development, hemimegalencephaly, nodular heterotopia, PTEN

## Abstract

Brain development and subsequent brain function are highly sensitive to genetic mutations, which can result in severe neurodevelopmental malformations. Alterations in PTEN signaling cause a spectrum of developmental malformations and neurological diseases including epilepsy. To date, a detailed understanding of the neuropathological underpinnings of *PTEN*‐associated brain malformations, particularly in fetuses, is missing. We have thus investigated a fetal case of hemimegalencephaly (HME), which is a rare disorder characterized by hemispheric overgrowth, developmental delay, and epileptic seizures. Our assessment of the male fetus includes genetic, radiologic, and histologic features and provides a comprehensive characterization of the cellular alterations in HME together with a genotypic correlation. Genetic analyses uncovered that hemispheric overgrowth was caused by a somatic second hit resulting in biallelic *PTEN* alteration in the affected brain tissue, although the unaffected hemisphere carried the same *PTEN* variant as the heterozygous germline variant. Based on the latter, we interpret that the *PTEN* mutation is not a dominant‐negative variant. Within the outer subventricular zone of the enlarged cortex, we found small nodular heterotopias, which can be origins of focal epileptic seizures. Cell type‐specific marker stainings revealed that the heterotopias consisted exclusively of SATB2^+^ glutamatergic projection neurons. Altogether, our analyses and findings contribute to a deeper understanding of the pathomechanisms of a novel *PTEN* variant driving a severe brain malformation.

**Key points**

- Upon biallelic alteration, a novel *PTEN* variant is associated with hemispheric overgrowth during fetal brain development.
- The affected hemisphere presents with focal nodular heterotopias within the outer subventricular zone.
- *PTEN* variant‐associated heterotopias consist of SATB2^+^ neurons.

## 1 INTRODUCTION

The brain comprises a remarkable cell‐type diversity, which arises during embryonic and fetal development and provides the basis for adequate brain function. Errors in the tightly orchestrated process of brain development, for example, by generating distinct subtypes of neurons in wrong ratios, can result in neurodevelopmental malformations and neurological diseases including epilepsy.^1^

Alterations in PTEN signaling, an essential regulator of cell growth, proliferation, survival, and metabolic activity, are known to cause brain malformations and neurological diseases of developmental origin. Depending on the time point/cell type in which respective mutations occur, altered PTEN signaling results in a spectrum of defects ranging from focal cortical dysplasia to hemimegalencephaly (HME) and macrocephaly.^2^, ^3^, ^4^ HME is a rare *PTEN*‐associated neurodevelopmental disorder,^5^, ^6^ characterized by overgrowth of one hemisphere, developmental delay, and epileptic seizures.^7^ However, a detailed cellular and molecular understanding of the neuropathological underpinnings of HME and onset of neurological symptoms, particularly in fetuses, is still missing to date.

We have thus assessed the genetic, radiologic, and histologic features of a pronounced HME in a male fetus (gestational week [GW] 22) and provide a comprehensive characterization of the cellular alterations in this malformation, focusing on the cortex and the locally produced glutamatergic neurons. HME provides the unique opportunity to directly compare the unaffected and megalencephalic hemispheres derived from the same developmental time point, harboring the same gross genetic background, and that have undergone identical postmortem processing for magnetic resonance imaging (MRI), genetic, and histological analysis.

## 2 RESULTS

### 2.1 Hemisphere‐specific loss of heterozygosity of a novel *PTEN* variant

**FIGURE 1:**
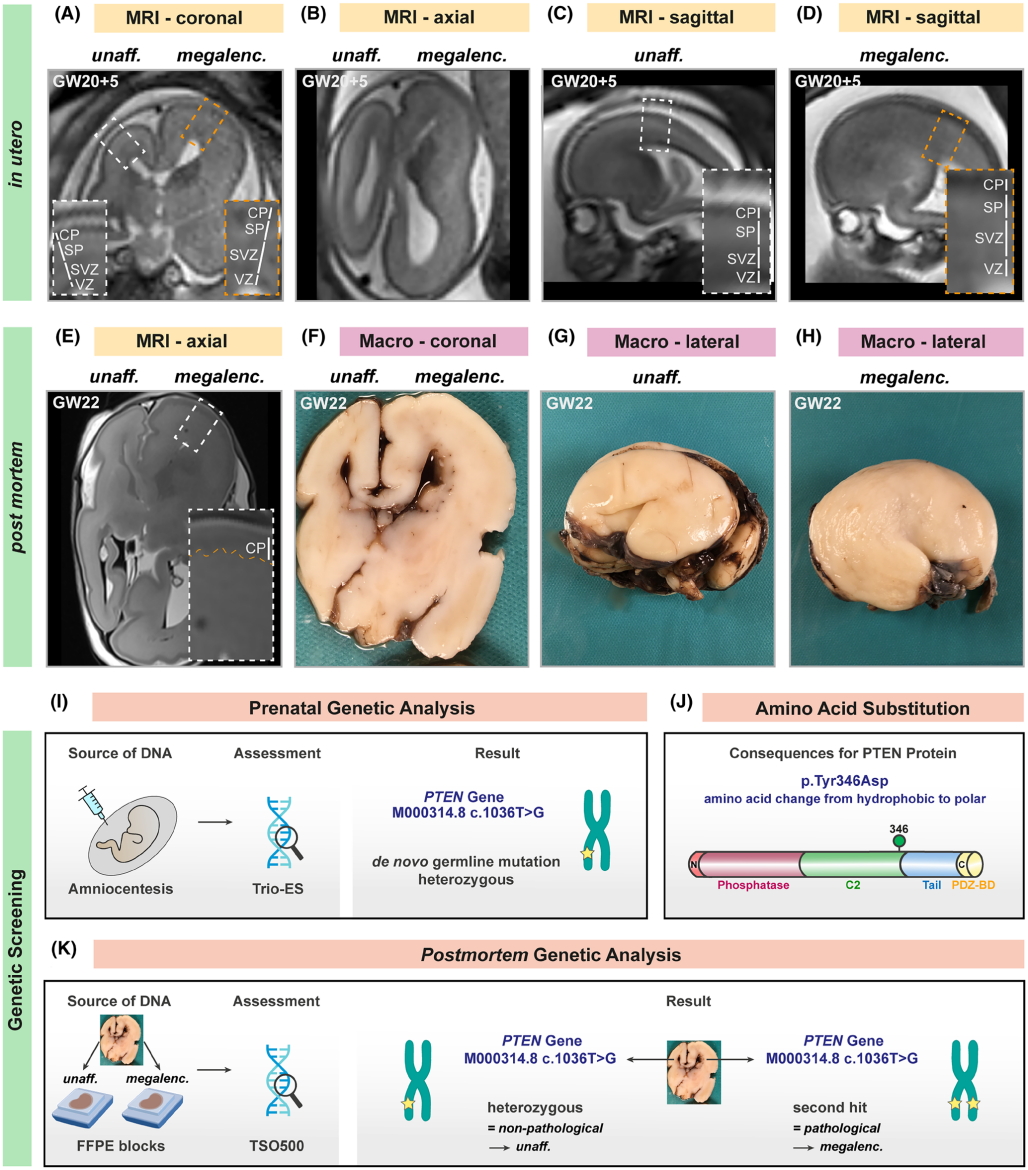
Novel pathological *PTEN* variant in a fetus with hemimegalencephaly. (A–D) In utero evaluation. Selected coronal (A, B) and sagittal (C, D) T2‐weighted images from 1.5‐T in utero MRI examination of a fetus with hemimegalencephaly at gestational week (GW) 20 + 5 are shown. Distinct cortical zones can be discriminated based on signal intensity in the zoom‐ins. The enlarged right hemisphere shows an almost homogeneous signal across all cortical zones, thus not allowing discrimination between particular zones such as the ventricular zone (VZ), the subventricular zone (SVZ), the subplate (SP), and the cortical plate (CP). (E–H) Postmortem evaluation. (E) T2‐weighted axial image of 3‐T postmortem magnetic resonance imaging (MRI) of the fetal brain at GW 22, correlating with the observations gained from (F) macroscopic coronal section of the same brain. (G, H) Postmortem whole‐mount photographs of the lateral view of the fetal brain, correlating well with the in utero MRI findings shown in panels C and D. (I–K) Genetic evaluation. (I) Results from trio exome sequencing (ES) following amniocentesis identified a heterozygous de novo point variant in *PTEN* (c.1036 T>G). (J) Schematic of the PTEN protein, indicating the location of the amino acid substitution in the PTEN protein caused by the c.1036 T>G variant. (K) Results from TruSight Oncology 500 (TSO500) panel sequencing of DNA isolated from the formalin‐fixed hemispheres. FFPE, formalin‐fixed paraffin‐embedded; megalenc., megalencephaly; unaff., unaffected; PDZ‐BD, PDZ binding domain motif.

In utero fetal MRI (1.5 T) was performed at GW 20 + 5 to confirm the ultrasonographic suspicion of a unilateral and asymmetric enlargement of the entire right hemisphere with cortical dyslamination, as determined by loss of the normal T2‐high signal within the subplate (SP) zone (Figure 1A–D).^8^ Pregnancy was terminated at 22 weeks of gestation. A postmortem brain MRI (3 T) showed similar findings as in utero MRI (Figure 1E). In addition, subtle areas of irregular and undulating cortical plate (CP) surface, mainly along the medial frontobasal and medial occipital lobes, were noticed (Figure 1E). Both MRI examinations correlated with the gross macroscopic appearance of the brain, showing a similar pronounced enlargement and irregular surface of the right hemisphere as compared to the smooth and normal‐sized left hemisphere (Figure 1F–H).

To perform a phenotype–genotype correlation, we first turned to the prenatal trio exome sequencing (ES) results, which were obtained after amniocentesis (Figure 1I). Trio ES identified a novel heterozygous de novo point variant in the phosphatase and tensin homolog (*PTEN*) gene (NM_000314.8: c.1036 T>G; Figure 1I), causing an amino acid substitution in the C2 domain of the PTEN protein (p.Tyr346Asp; Figure 1J). Because PTEN serves as a negative regulator of mTOR signaling, *PTEN* loss‐of‐function variants typically result in overgrowth syndromes.^3^, ^4^, ^5^, ^6^ However, a dominant‐negative germline variant in *PTEN* should result in a whole‐body overgrowth. Instead, the fetus showed no signs of peripheral organ abnormalities and displayed normal body length as compared to healthy fetuses of the same developmental stage (Supplemental Material) but had a unilateral HME phenotype where only one hemisphere overgrew, thus suggesting the presence of a second somatic hit or synergizing mutations in the affected hemisphere.^9^

To uncover hemisphere‐specific somatic mutations, we isolated DNA from formalin‐fixed paraffin‐embedded brain tissue of the unaffected and the affected hemisphere and performed next generation sequencing using TruSight Oncology 500 panel sequencing (Figure 1K, Table S1). In the unaffected hemisphere, we found an allelic frequency of 49.31% for the *PTEN* c.1036 T>G variant, thus reflecting the heterozygous germline mutation that was already discovered by trio ES (Figure 1K). Importantly, the megalencephalic hemisphere showed an allelic frequency of 88.06% for the *PTEN* c.1036 T>G variant, consistent with loss of heterozygosity in the neuronal lineage (Figure 1K, Table S1). As a result, we assume that the neuronal lineage of the megalencephalic hemisphere carries the *PTEN* c.1036 T>G variant in a biallelic manner, and that other lineages, such as endothelial cells and immune cells, carry the heterozygous germline variant. We thus refer to the unaffected hemisphere as *PTEN* ^*+/−*^ and to the megalencephalic hemisphere as *PTEN* ^*−/−*^.

### 2.2 Biallelic *PTEN* variant causes reduced PTEN signaling and nodular heterotopias in the overgrown hemisphere

**FIGURE 2:**
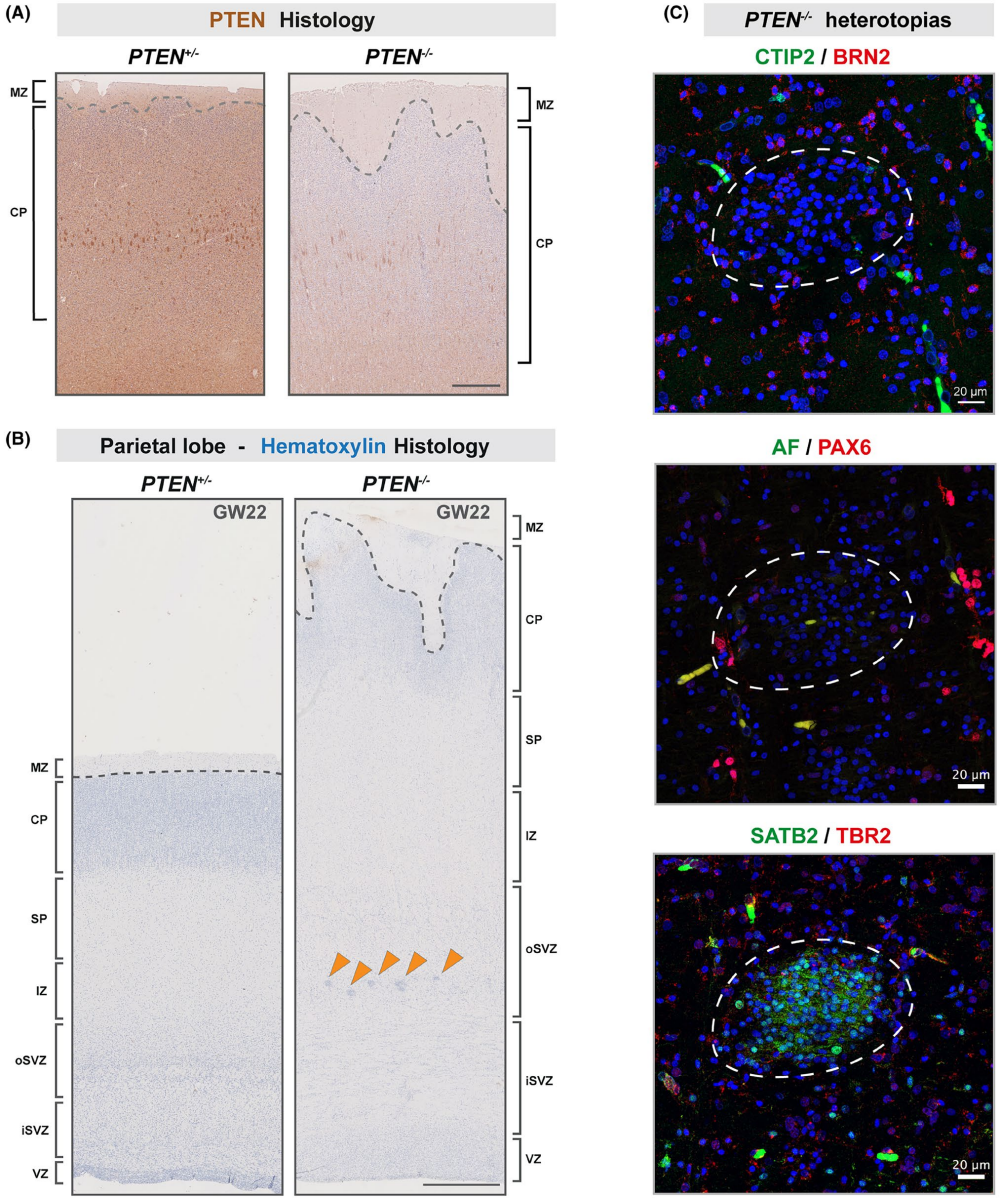
Histological appearance of *PTEN*‐associated hemimegalencephaly. (A) PTEN staining of the unaffected (*PTEN* ^*+/−*^) and the affected hemisphere (*PTEN* ^*−/−*^). (B) Hematoxylin staining of the unaffected and the *PTEN* ^*−/−*^ hemisphere and identification of the different zones of the fetal cortex. Orange arrowheads highlight nodular heterotopias localized in the outer subventricular zone (oSVZ) of the *PTEN* ^*−/−*^ hemisphere. (C) High‐resolution images of immunofluorescent staining of representative nodular heterotopia, showing (top panel) layer V neuron marker CTIP2 (green) and layer II–III neuron marker BRN2 (red), (middle panel) apical radial glial progenitor marker PAX6 (red) and autofluorescence (AF; green), and (lower panel) intermediate progenitor marker TBR2 (green) and layer II–IV neuron marker SATB2 (red), together with nuclear stain (4,6‐diamidino‐2‐phenylindole, blue). Scale bars for panels A and B: 250 μm; scale bars for panel C: 20 μm. CP, cortical plate; GW, gestational week; iSVZ, inner subventricular zone; IZ, intermediate zone; MZ, marginal zone; SP, subplate; VZ, ventricular zone.

To validate our hypothesis of the biallelic *PTEN* c.1036 T>G variant resulting in reduced PTEN levels/activity, we performed PTEN immunostaining. The biallelic variant resulted in strongly diminished PTEN immunoreactivity (Figure 2A), which correlated with tissue enlargement (Figures 1 and 2B) and was in line with the known role of *PTEN* loss of function in overgrowth syndromes.^9^ Based on these results we infer that the c.1036 T>G variant is pathogenic upon biallelic alteration. According to the American College of Medical Genetics and Genomics criteria, we thus classify the variant as “likely pathogenic” (Table S1).^10^

Next, we used nuclear staining with hematoxylin on histological sections to discriminate between the different zones of the developing fetal neocortex based on cell density (according to the developing human brain atlas; Figure 2B).^11^, ^12^ We detected nodular heterotopias specifically located in the outer subventricular zone (oSVZ) of the megalencephalic hemisphere (arrowheads, Figure 2B). The heterotopias were not visible on MRI, likely reflecting limited sensitivity at this developmental stage. Additionally, the CP of the *PTEN* ^*−/−*^ hemisphere showed features of cortical dyslamination and an irregular surface with many fingerlike protrusions, which correlated well with the MRI observations (Figures 1E and 2B).

To gain a deeper understanding of the nodular heterotopias occurring in *PTEN*‐associated HME, we stained for cell type‐specific markers (Figure 2C), using anti‐PAX6 antibody to identify apical radial glial progenitors and anti‐TBR2 antibody for intermediate progenitors.^13^, ^14^ Cortical projection neurons were discriminated based on CTIP2 (early born subcerebral neurons located in layer V), SATB2 (late born callosal neurons in layers II–IV), and BRN2 (late born corticocortical neurons in layer II–III) immunostaining.^15^, ^16^ We could not detect any progenitor cell subtypes within the heterotopias as determined by absence of PAX6^+^ or TBR2^+^ cells, suggesting that these ectopic cells consisted of neurons. We found the heterotopias to almost exclusively harbor upper layer glutamatergic SATB2^+^ cells and not lower layer CTIP2^+^ or upper layer BRN2^+^ cells (Figure 2C). Further immunohistochemistry stainings with interneuron markers (calbindin, calretinin, GAD67, somatostatin; Figure S1)^17^ showed that the heterotopias did not display interneuron enrichment, thus strengthening the evidence of a glutamatergic lineage phenotype in the *PTEN^−/−^* hemisphere.

## 3 DISCUSSION

Our study provides novel insights into the asymmetric genetic and cellular alterations occurring in a case of HME. Many *PTEN*‐associated diseases are caused by dominant‐negative *PTEN* variants.^18^ However, here we describe a new *PTEN* c.1036 T>G variant and unravel its pathogenicity in the biallelic state directly from the affected tissue by means of next generation sequencing.

Although a heterozygous germline variant could be responsible for a more systemic phenotype, the fetus did not present with further pathologies other than the pronounced hemimegalencephaly (see autopsy report in the Supplemental Material). We thus interpret that the heterozygous germline variant is not pathogenic and therefore does not cause a systemic phenotype, at least at this developmental stage.

The megalencephalic hemisphere showed an irregular cortical plate/marginal zone boundary. A recent study using cerebral organoids was able to show that a pathogenic *PTEN* loss‐of‐function mutation increased the proliferation potential of cortical progenitors, inducing expansion and folding during corticogenesis.^19^ The folded organoids contained expanded ventricular zone (VZ) and oSVZ progenitors, as well as delayed neural differentiation,^19^ similar to what we found in the megalencephalic hemisphere of the presented fetus.

The oSVZ of the *PTEN* ^*−/−*^ cortex contains small focal heterotopias, which consist of SATB2^+^ glutamatergic neurons. Neuronal nodular heterotopias are a source of altered neuronal circuits causing focal seizure activity^20^ and have been described in HME before.^21^ However, heterotopia cell composition may vary in a patient‐ and/or *PTEN* variant‐specific manner. Whereas we identified mainly a glutamatergic neuronal phenotype in the fetus, as determined by the presence of SATB2^+^ neurons in the nodular heterotopias, Hannan et al.^21^ found an enrichment of γ‐aminobutyric acidergic interneurons in the heterotopias of four postnatal HME cases. This hypothesis is supported by a recent report on a case of HME with epilepsy, harboring distinct *PTEN* variants in different regions of the cortex and showing variant‐specific alterations of the cellular composition in affected cortical areas.^9^ Because we did not perform ultradeep sequencing as reported by Sanders et al.,^20^ we cannot formally rule out that heterotopias may consist of genetically distinct cells with additional low‐level somatic pathogenic variants (allelic frequency <5%). Yet, the expansion of the cortical VZ and oSVZ, as well as our histological assessment of glutamatergic and inhibitory neurons, provides strong evidence that the presented case mainly harbors a glutamatergic lineage phenotype.

Importantly, the heterotopias in the *PTEN* ^*−/−*^ fetal hemisphere could not be detected using MRI. Yet, they were clearly visible in histological sections. Our data thus underline the necessity to study brain malformations with both radiological and histological methods for a precise integrated diagnosis.

## AUTHOR CONTRIBUTIONS

Franziska Fazekas and Nicole Amberg designed the research project, performed histological analyses, and coordinated data collection. Bernhard Hennebichler coordinated the DNA isolation from formalin‐fixed paraffin‐embedded tissue and subsequent TruSight Oncology 500 panel sequencing. Thomas Roetzer‐Pejrimovsky performed TSO500 result interpretation. Amit Haboosheh, Christian Mitter, and Gregor Kasprian performed MRI assessment. Christof Worda, Julia Binder, Theresa Reischer, Tina Linder, and Alex Farr performed the clinical patient counseling. Mateja Pfeifer coordinated the trio ES screening of DNA isolated following amniocentesis. Anke Scharrer performed the fetal autopsy. Romana Höftberger, Ellen Gelpi, and Christine Haberler performed the diagnostic neuropathological examinations and provided neuropathological expertise. Franziska Fazekas, Amit Haboosheh, and Nicole Amberg wrote the manuscript with input from all coauthors. Franziska Fazekas and Nicole Amberg prepared the figures with input from all coauthors.

## FUNDING INFORMATION

N.A. received funding from the FWF (Elise Richter program, grant V1041T).

## CONFLICT OF INTEREST STATEMENT

The authors have no competing interests. We confirm that we have read the Journal's position on issues involved in ethical publication and affirm that this report is consistent with those guidelines.

## ETHICS APPROVAL

The present study was approved by the ethics committee of the Medical University of Vienna (EK 1161/2018). This study is part of the Neurobiobank of the Division of Neuropathology and Neurochemistry, Department of Neurology, Medical University of Vienna. Research use of biobanked samples was approved by the ethics committee of the Medical University of Vienna (EK 1636/2019), which provides a common broad consent (biobank consent) according to the Austrian Research Organization Act 2018, §2d, para 3.

## CONSENT FOR PUBLICATION

Family members have consented to publication as part of their consent for tissue archiving in our neurobiobank (EK 1636/2019).

## Supporting information


DATA S1.

TABLE S1.

## Data Availability

The trio ES data that support the findings of this study are available on request from the corresponding author. The data are not publicly available because of privacy or ethical restrictions.
