# Supplementary material for: A novel PTEN variant causing hemimegalencephaly and focal nodular heterotopias in the developing human brain
Source: Epilepsia. 2026 Jan 5;67(2):e32–8. doi: 10.1002/epi.70088 (PMC12927695; doi:10.1002/epi.70088)
Supplement: Supplementary file 1 — DATA S1. [file EPI-67-e32-s002.docx]

**Supplemental Material**

**A novel *PTEN* variant causing hemimegalencephaly and focal nodular heterotopias in the developing human brain**

**Authors**

Franziska Fazekas^1,6, *^, Amit Haboosheh^2,6,7 *^, Bernhard Hennebichler^1,6^, Thomas Roetzer-Pejrimovsky^1,6^, Julia Binder^3^, Theresa Reischer^3^, Mateja Pfeifer^4^, Anke Scharrer^5^, Christof Worda^3^, Tina Linder^3^, Alex Farr^3^, Romana Höftberger^1,6^, Ellen Gelpi^1,6^, Christian Mitter^2,6^, Gregor Kasprian^2,6^, Christine Haberler^1,6^, Nicole Amberg^1,6 #^

**Affiliations**

^1^ Medical University of Vienna, Department of Neurology, Division of Neuropathology and Neurochemistry, Vienna, Austria

^2^ Medical University of Vienna, Department of Biomedical Imaging and Image-guided Therapy, Division of Neuroradiology and Musculoskeletal Radiology, Vienna, Austria

^3^ Medical University of Vienna, Department of Obstetrics and Gynecology, Division of Obstetrics and Feto-Maternal Medicine, and Comprehensive Center for Pediatrics (CCP), Vienna, Austria

^4^ Medical University of Vienna, Center for Pathobiochemistry and Genetics, Institute of Medical Genetics, Vienna, Austria

^5^ Medical University of Vienna, Department of Pathology, Vienna, Austria

^6^ Comprehensive Center for Clinical Neurosciences & Mental Health, Medical University of Vienna, Vienna, Austria

^7^ Hadassah Medical Center, Department of Radiology, Jerusalem, Israel

^*^ These authors have equally contributed to the manuscript

^#^ Corresponding author

**Summary of the examinations performed on the presented fetal case with hemimegalencephaly**

**Sonographic measurements performed by the Division of Obstetrics and Feto-Maternal Medicine, Medical University of Vienna**

| **parameter** | **GW20+2 (*in utero*)** | **GW 21+4 (*in utero*)** |
| --- | --- | --- |
| BPD (biparietal diameter): | 57.9mm | 63.5mm |
| FOD (fronto-occipital diameter): | 83.4mm | 89.7mm |
| HC (head circumference): | 224mm | 242.4mm |
| percentile | >97 | >97 |

**MRI measurements performed by the Division of Neuroradiology, Medical University of Vienna**

| **parameter** | **GW20+5 (*in utero*)** |
| --- | --- |
| Fetal length (crown-rump-length): | 180mm |
| HC (head circumference): | 230mm |

**General autopsy report by the Department of Pathology, Medical University of Vienna**

**Age:** GW 22

**Sex:** male

**Body length:** 31 cm

**Body weight:** 610g

**Detected diseases during pregnancy:**

Hemimegalencephaly

**Procedures/Examinations:**

IVF: no

Invasive diagnostics: yes (amniocentesis)

MRI *in utero*: yes

MRI *postmortem*: yes

Native X ray: no

Fibroblast culture: no

Placenta examination: yes, appeared normal

Umbilical cord examination: yes, appeared normal

**APGAR score:**

0/0/0

**Pathological examination and macroscopic evaluation:**

Cerebral malformation

Hypertelorism

Retrognathia

No primary extracerebral organ malformation

**Organ weight [g]:**

Brain: 202 (of which 3.8g are cerebellum and brainstem).

Heart: 3,29

Liver: 25,49

Spleen: 0,73

Kidney left: 2,26

Kidney right: 1,90

Lung left: 6,55

Lung right: 7,84

Adrenal gland left: 1,26

Adrenal gland right: 1,01

Thymus: 0,67

**Gross brain examination report by the Division of Neuropathology and Neurochemistry, Medical University of Vienna**

The non-dissected, 10% formalin-fixed fetal brain was transferred from the Department of Pathology to the Division of Neuropathology and Neurochemistry (Medical University of Vienna). Following three weeks of fixation, brain autopsy was performed.

The total brain weight was 202 g, of which the cerebellum and brainstem weight was 3.8 g (at this gestational age, a total weight of 78 +/- 14 g would be expected; with a normal infratentorial/cerebellum and brainstem weight of 3.7 +/- 0.7 g).

The fronto-occipital diameter on the left measured 76 mm and the right 87 mm, the biparietal diameter measured 23 mm, and the cerebellum transversely measured 25 mm. Please note the differences with sonography due to the different tissue conditions.

External examination revealed moderate blood infiltration of the leptomeninges. At first glance, the right cerebral hemisphere was noticeably enlarged compared to the left. The brain surface on the left was smooth and shiny, while on the right side, the surface appeared somewhat duller and creased, with slight indentations running parallel to the interhemispheric fissure, resembling small apparent furrows.

There was no noticeable difference in tissue consistency between the two cerebral hemispheres. The lateral sulcus was present on both sides. Examination of the brain base revealed the presence of the olfactory bulbs, which were also significantly enlarged on the right side compared to the left. The optic chiasm was extremely delicate on both sides and not asymmetrical. In the infratentorial structures, there was a slight asymmetry of the cerebellar hemispheres, possibly slightly enlarged on the right side, although the difference was significantly less than in the cerebral region.

Frontal sections through the cerebrum confirmed the strong hemispheric asymmetry, with a significant increase in volume and thickening of the entire right cerebral parenchyma, including the cerebral mantle, the primordium of the anterior basal ganglia, the middle regions, and also the thalamus, which was enlarged compared to the left side. The entire temporal lobe and the right hippocampus were enlarged and plump compared to the left side. Medially, in the cingulate region, an asymmetrical fold with delicate furrowing was evident on the right side, while being absent on the left side. A similar pattern was also seen on the right occipito-medial side, where medial furrows were evident, which were not visible on the left side. The left hemisphere showed a largely normal development of all structures, although it may have been slightly enlarged for the given gestational age. The lateral ventricles on both sides were dilated, particularly in the posterior segments. No parenchymal or intraventricular hemorrhages were detected. No hardening was observed on the cut surface, and no calcifications were macroscopically visible.

Sections through the cerebellum and brainstem gave the impression of slight enlargement of the right pontine base; the pyramidal tract may not have been clearly asymmetrical macroscopically due to the sectioning.

The cerebellar hemisphere on the right side also appeared somewhat enlarged compared to the left side, especially in the posterior segments. In contrast, the left cerebellar hemisphere may have appeared slightly bulged in the anterior segments, which could simply be due to the sectioning. The vermicular area was macroscopically present and easily identifiable in all segments.


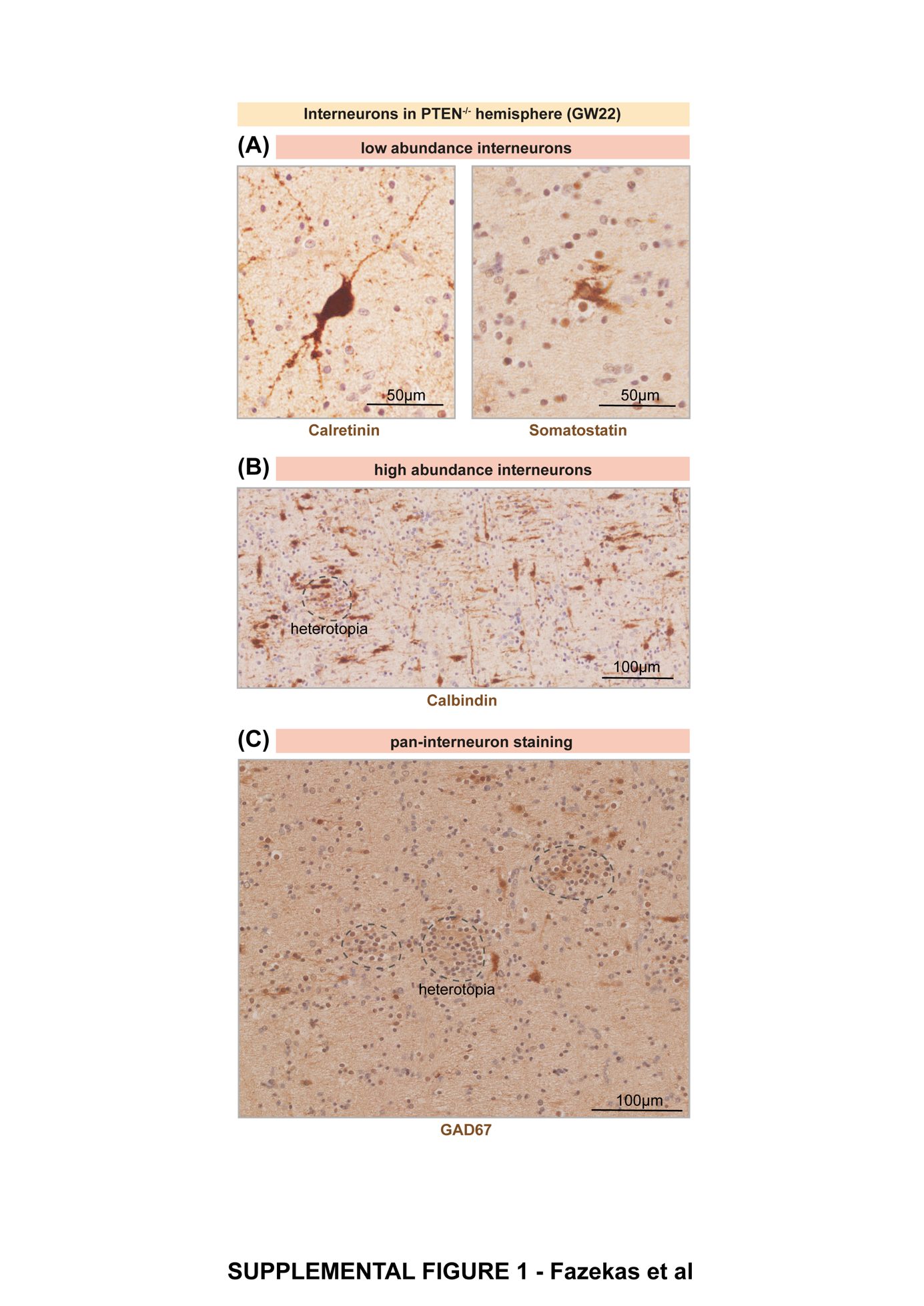


**Supplemental Figure 1: Focal heterotopias do not show interneuron enrichment**

**A-C)** **Interneuron stainings.** Representative images of **(A)** specific low abundance interneurons (Calbindin positive (left), Somatostatin positive, right), **(B)** specific high abundance interneurons (Calretinin positive), and **(C)** pan-interneuron marker GAD67. None of the interneuron populations show an enrichment in the focal heterotopias, which are indicated by dashed lines. Scale bars in (A): 50µm, in (B-C) 100µm.
