# Supplementary material for: A novel PTEN variant causing hemimegalencephaly and focal nodular heterotopias in the developing human brain
Source: Epilepsia. 2026 Jan 5;67(2):e32–8. doi: 10.1002/epi.70088 (PMC12927695; doi:10.1002/epi.70088)
Supplement: Supplementary file 2 — TABLE S1. [file EPI-67-e32-s001.docx]

| **TSO500 results GW22 fetal cortex** | |  |  |  |  |  |
| --- | --- | --- | --- | --- | --- | --- |
|  |  |  |  |  |  |  |
|  | **unaffected hemisphere** | | | | | |
|  | **gene** | **variant** | **chromosome** | **type and effect** | **allelic frequency** | **evaluation** |
| **shared loci** | *FANCA* p.Ml? | ENST0000038930 l.3 c.2T>C | 16 | SNV, start loss | 36.49 | likely oncogenic / pathogenic |
|  | *CTCF* p.G585= | ENST00000264010.4 c.1755G>A | 16 | SNV, silent | 56.73 | uncertain significance |
|  | *FANCI* | ENST00000310775.7 c.3721-5C>T | 15 | SNV, splice site extended | 50.13 | uncertain significance |
|  | *FUBP1* | ENST00000370768.2 c.1151C>A | 1 | SNV missense | 56.68 | uncertain significance |
|  | *GPSM3* | ENST00000375040.3 c.-3014G>A | 6 | SNV 5' flankig | 45.05 | uncertain significance |
|  | *NAB2* | ENST00000300131.3 c.*288C>G | 12 | SNV 3' UTR | 40.97 | uncertain significance |
|  | *PDK1* p.Nl76= | ENST00000282077.3 c.528T>C | 2 | SNV silent | 51.91 | uncertain significance |
|  | ***PTEN* p.Y3460** | ENST00000371953.3 c.1036T>G | 10 | SNV missense | **49.31** | likely pathogenic |
|  | *SMC3* p.Q230H | ENST00000361804.4 c.690G>C | 10 | SNV missense | 57.88 | uncertain significance |
|  | *SPIRE2* | ENST00000378247.3 c.-11937A>G | 16 | SNV 5' flankig | 36.49 | uncertain significance |
|  | *SPTA1* p.L266= | ENST00000368147.4 c.798A>G | 1 | SNV silent | 59.07 | uncertain significance |
|  | *STAT6* | ENST00000300134.3 c.*1563G>C | 12 | SNV 3' flanking | 40.97 | uncertain significance |
|  | *PIK3C2B* p.L202R | ENST00000367187.3 c.597_605delinsAGGCAAACG | 1 | MNP missense | 48.41 | uncertain significance |
|  | *BRAF* | ENST00000288602.6 c.1993-11dup | 7 | ins splice site | 47.37 | uncertain significance |
|  | *ZBTB7A* copy number gain |  | 19 | copy gain |  | unclassified |
| **hemisphere-specific loci** | *FLI* | ENST 00000527786.2 C1833_*1834insCTCAATTT TGAAATCTGCAAAGTATTCAT TAGGGAAAAT | 11 | ins 3' UTR | 32.26 | uncertain significance |
|  | *GNA13* copy number gain | - | 17 | copy gain | - | unclassified |

|  | **megalencephalic hemisphere** | | | | | |
| --- | --- | --- | --- | --- | --- | --- |
|  | **gene** | **variant** | **chromosome** | **type and effect** | **allelic frequency** | **evaluation** |
| **shared loci** | *FANCA* p.Ml? | ENST0000038930 l.3 c.2T>C | 16 | SNV, start loss | 40.44 | likely oncogenic / pathogenic |
|  | *CTCF* p.G585= | ENST00000264010.4 c.1755G>A | 16 | SNV, silent | 50.22 | uncertain significance |
|  | *FANCI* | ENST00000310775.7 c.3721-5C>T | 15 | SNV, splice site extended | 46.76 | uncertain significance |
|  | *FUBP1* | ENST00000370768.2 c.1151C>A | 1 | SNV missense | 47.31 | uncertain significance |
|  | *GPSM3* | ENST00000375040.3 c.-3014G>A | 6 | SNV 5' flankig | 45.39 | uncertain significance |
|  | *NAB2* | ENST00000300131.3 c.*288C>G | 12 | SNV 3' UTR | 47.84 | uncertain significance |
|  | *PDK1* p.Nl76= | ENST00000282077.3 c.528T>C | 2 | SNV silent | 52.46 | uncertain significance |
|  | ***PTEN* p.Y3460** | ENST00000371953.3 c.1036T>G | 10 | SNV missense | **88.06** | likely pathogenic |
|  | *SMC3* p.Q230H | ENST00000361804.4 c.690G>C | 10 | SNV missense | 87.21 | uncertain significance |
|  | *SPIRE2* | ENST00000378247.3 c.-11937A>G | 16 | SNV 5' flankig | 40.44 | uncertain significance |
|  | *SPTA1* p.L266= | ENST00000368147.4 c.798A>G | 1 | SNV silent | 49.66 | uncertain significance |
|  | *STAT6* | ENST00000300134.3 c.*1563G>C | 12 | SNV 3' flanking | 47.84 | uncertain significance |
|  | *PIK3C2B* p.L202R | ENST00000367187.3 c.597_605delinsAGGCAAACG | 1 | MNP missense | 57.18 | uncertain significance |
|  | *BRAF* | ENST00000288602.6 c.1993-11dup | 7 | ins splice site | 45.76 | uncertain significance |
|  | *ZBTB7A copy number gain* |  | 19 | copy gain | - | unclassified |
| **hemisphere-specific loci** | *ANKRD11* p.A1780T | ENST00000301030.4 c.5338G>A | 16 | SNV missense | 40.72 | likely benign |
|  | *BCR* p.V949I | ENST00000305877.8 c.2845G>A | 22 | SNV missense | 43.88 | likely benign |
|  | *CARD11* p.I544L | ENST00000396946.4 C. 1630A>C | 7 | SNV missense | 46.38 | likely benign / benign |
|  | *EPHA3* p.A427= | ENST00000336596.2 c.1281G>A | 3 | SNV silent | 39.76 | likely benign |
|  | *MAP2K2* p.P282= | ENST00000262948.5 c.846C>T | 19 | SNV silent | 51.30 | likely benign |
|  | *NOTCH4* p.P1974= | ENST00000375023.3 C.5922G>A | 6 | SNV silent | 45.39 | likely benign |
|  | *PDGFRB* p.R502Q | ENST00000261799.4 c.1505G>A | 5 | SNV missense | 44.72 | likely benign |
|  | *PIK3C2B* p.L202R*. ** | ENST00000367187.3 C.605T>G | 1 | SNV missense | 57.18 | likely benign |
|  | *PPP2R2A* p.H2P | ENST00000315985.7 C.35A>C | 8 | SNV missense | 56.12 | likely benign / uncertain significance |
|  | *PRKC1* p.V299= | ENST00000295797.4 C.897A>C | 3 | SNV silent | 54.29 | likely benign |
|  | *TGFBR2* | ENST00000295754.5 c.94+16245G>A | 3 | SNV non-coding | 44.25 | likely benign |
|  | *TGFBR2* p.D40N | ENST00000359013.4 c.118G>A | 3 | SNV missense | 44.25 | likely benign |

| * Whether the megalencephalic hemisphere-specific SNV in PIK3C2B p.202R, ENST00000367187.3 C.605T>G is due to a technical inconsistency / limitation in the TSO500 panel sequencing is unclear |
| --- |

| **other PI3K / PTEN / mTOR pathway genes covered by the TSO500 panel, but not showing results on the input DNA from the presented fetal case:** | |
| --- | --- |
| *AKT1, AKT2, AKT3* | |
| *MTOR, RICTOR, RPTOR* | |
| *MAP2K1, MAP2K3, MAP2K4, MAP3K1, MAP3K4, MAP3K13, MAP3K14, MAPK1, MAPK3* | |
| *PIK3C2B, PIK3C2G, PIK3C3, PIK3CA, PIK3CB, PIK3CD, PIK3CG, PIK3R1, PIK3R2, PIK3R3* | |
| *RHEB* |  |
| *RPS6KA4, RPS6KB1, RPS6KB2* | |
| *TSC1, TSC2* |  |
